# An outbreak of *Salmonella* Saintpaul in a Scottish childcare facility: the influence of parental under-reporting

**DOI:** 10.1186/s12879-019-4516-z

**Published:** 2019-10-15

**Authors:** Rachel M. Thomson, Hazel J. Henderson, Alison Smith-Palmer

**Affiliations:** 1 Public Health Department (Afton House), NHS Ayrshire & Arran, Dalmellington Road, Ayr, KA6 6AB Scotland, UK; 2 Health Protection Scotland, Glasgow, Scotland, UK

**Keywords:** Salmonella, Outbreaks, Gastrointestinal disease, Public health, School outbreaks, Epidemiology

## Abstract

**Background:**

*Salmonella* outbreaks in childcare facilities are relatively rare, most often occurring secondary to contaminated food products or poor infection control practices. We report an outbreak of *Salmonella* Saintpaul at a pre-school facility in Ayrshire, Scotland with atypical clinical and epidemiological features.

**Methods:**

Following notification of the initial two cases, the multi-disciplinary Incident Management Team initiated enhanced active case finding and two environmental inspections of the site, including food preparation areas. Parent and staff interviews were conducted by the Public Health department covering attendance, symptomatology and risk factors for all probable and confirmed cases. Microbiological testing of stool samples and the facility water tank was conducted. Whole Genome Sequencing (WGS) was performed for positive stool samples at the national reference laboratory. Infection control measures were introduced iteratively due to the atypical progression of the outbreak.

**Results:**

There were 15 confirmed cases and 3 children admitted to hospital during the outbreak. However, 35.7% of cases reported extremely mild symptoms. The attack rate was 15.2%, and age of affected children ranged from 18 to 58 months (mean 35 months). All cases were the same Multilocus Sequence Type (MLST50). Epidemiological investigation strongly suggested person-to-person spread within the facility. Existing infection control practices were found to be of a high standard, but introduction of additional evidence-based control measures was inadequate in halting transmission. Facility staff reported concerns about lack of parental disclosure of gastrointestinal symptoms, particularly where these were mild, with 50.0% of cases having attended while symptomatic against public health advice. Voluntary two-week closure of the facility was implemented to halt transmission, following which there were no new cases. WGS results were unavailable until after the decision was taken to close the facility.

**Conclusions:**

This is the first reported instance of a *Salmonella* Saintpaul outbreak at a childcare facility, or where person-to-person transmission is indicated. Clinicians should consider the influence of parental under-reporting on gastrointestinal outbreaks in childcare settings, particularly where perceived severity is low and financial or social pressures to attend work may reduce compliance. WGS cannot yet replace conventional microbiological techniques during short, localised outbreaks due to delays receiving results.

## Background

*Salmonella* is a ubiquitous bacterium with more than 2000 serotypes identified [1]. However, most disease in Scotland is caused by either *S.* Enteritidis or *S.* Typhimurium, with *S.* Saintpaul being a rarer serotype accounting for only 3% of Scottish cases in 2017 (a majority of whom were part of the reported outbreak). The total number of confirmed *Salmonella* cases in Scotland has fallen over time from 1029 in 2006 to 838 in 2017 [2].

Transmission occurs by ingestion of contaminated food (most commonly red meat, poultry, raw eggs, and dairy products) or by faecal contamination from an infected person or animal e.g. in contaminated water [1]. Incidence is higher in infants and young children [3]. The usual incubation period is 6–72 h, though it is known that this can be prolonged where the bacterial dose ingested by individuals is small [4]. The period when an individual is infectious to others varies, with most cases excreting the bacteria in faeces for several days to several weeks. A temporary carrier state may continue for months, especially in infants [3]. While most cases are self-limiting and involve mild to moderate gastrointestinal (GI) symptoms, more serious complications such as bacteraemia or meningitis can occur in rare cases [5].

Outbreaks of *Salmonella* infection in childcare facilities are relatively rare, most often occurring due to ingestion of contaminated food products or poor infection control practices [6–10]. Less common potential point sources for outbreaks include contamination of the wider environment, such as water or sand [11, 12]. Current UK-wide infection control guidance for schools and other childcare facilities states that any children or staff members diagnosed with *Salmonella* should be excluded until 48 h after symptoms have stopped [13].

All human serotypes of *Salmonella* are currently classed as notifiable organisms under the Public Health etc. (Scotland) Act 2008 [14]. There are 14 Scottish NHS health boards, each with their own Health Protection Team (HPT) located within the Public Health department who are responsible for investigating and managing all cases or associated incidents. Positive local laboratory results for *Salmonella* are reported by a Consultant Microbiologist to the local HPT and specimens sent to the national reference laboratory for confirmation and typing. Whole genome sequencing (WGS) is carried out, and isolates are reported by serotype. Prior to WGS results, antibiotic resistance and antigen agglutination profiles are available from local laboratories without serotyping.

In December 2017 the HPT in NHS Ayrshire and Arran was notified of two 2-year old children attending the same local pre-school childcare facility whose stool samples had tested positive for *Salmonella*. Dates of symptom onset were within 3 days of each other, and both children had been unwell enough to attend hospital with diarrhoea and dehydration. On preliminary local typing the *Salmonella* strains had the same antibiotic resistance profile and agglutinated with the same O4 antigen, indicating a reasonably high probability the strains were the same. On contacting the facility the HPT were informed of a third possible case, also 2 years old, who became symptomatic within the same period. At this point this incident was declared an outbreak and a multi-disciplinary Incident Management Team (IMT) was formed involving representatives from the childcare facility, local Public Health, Microbiology and Environmental Health, and national body Health Protection Scotland (HPS).

### Description of childcare facility

The facility in question has a wide catchment area and accepts children aged 0–5 years, split into three separate rooms of the building by age (0–1 years, 2–3 years and 4–5 years). At the time of the incident there were 92 children regularly attending, and 19 permanent staff members including administrative staff. The only mixing of children occurred for the small number who arrived between 7.30 am and 8.30 am (< 10), after which all activities (including eating and play) were undertaken separately by age cohort.

Childcare staff were assigned to individual rooms, but there was some cross-cover between rooms during staff breaks. There was one kitchen, with one dedicated chef for the whole facility. There was water and sand play inside each main room of the facility, and mud play in an outdoor garden area.

## Methods

### Case definitions

Case definitions for use during the outbreak were established following discussion among the HPT and wider IMT (Table 1).

**Table 1:** Final case definitions used throughout the outbreak of *S.* Saintpaul at a childcare facility in Ayrshire, December 2017

Case Definitions	
Possible case	Symptoms consistent with salmonellosis (nausea, vomiting, diarrhoea and/or abdominal pain) with date of onset from 23rd November to 22nd December and regular attendance at childcare facility in question without clear link to confirmed case.
Probable case	Symptoms consistent with salmonellosis (nausea, vomiting, diarrhoea and/or abdominal pain) with date of onset from 23rd November to 22nd December and regular attendance at childcare facility in question with clear link to confirmed case e.g. known to have attended on same day/in same room of facility; OR Symptoms consistent with salmonellosis (nausea, vomiting, diarrhoea and/or abdominal pain) with date of onset from 23rd November to 22nd December in household contact of probable or confirmed case.
Confirmed case	Laboratory-confirmed *Salmonella* infection with date of onset from 23rd November to 22nd December and regular attendance at childcare facility in question; OR Laboratory-confirmed *Salmonella* infection with date of onset from 23rd November to 22nd December in household contact of probable or confirmed case.

### Active case finding

Daily telephone communication between the childcare facility and the HPT was established to record any new possible or probable cases. Letters and a text reminder were distributed to parents and/or guardians of all children registered as attending the facility to alert them to the outbreak, improve reporting of symptoms and uncover historic cases. Signage was placed on the facility door to remind parents of the requirement to report any illness occurring at home, particularly GI illness.

Written communication was sent to all General Practitioners (GPs) in the area to alert them to the outbreak and advise them to identify samples from symptomatic children as associated with the childcare facility when submitting them to the laboratory.

### Environmental investigations

Two site visits were carried out to review infection control procedures and practices at the childcare facility, the first an unannounced inspection by the local Environmental Health department and the second by the HPT and an infection control specialist from HPS. The first visit involved a food safety inspection of the facility’s one kitchen and review of the menus for the previous 3 weeks. The same infection control specialist from HPS also conducted a telephone interview with the facility’s external cleaners to discuss their usual practice. Visual inspection of the water tank used by the facility was carried out by Environmental Health Officers.

### Epidemiological investigations

Parents and/or guardians of all possible or probable cases were contacted by the HPT and interviewed to determine date of onset, symptoms, any linked household cases, and possible risk factors. In addition, detailed information was gathered on their attendance at the facility in the preceding 2 weeks, including which days they had attended, which room(s) of the facility they had been in, and any days they attended after developing GI symptoms. On confirmation of *Salmonella* infection, a standardized gastrointestinal surveillance form was completed by a local Environmental Health Officer at a household visit for all cases and reviewed by the HPT.

All epidemiological data on cases were collated in the local health protection electronic record system and in Microsoft Excel.

### Laboratory investigations

Stool sample processing at the local laboratory involved sample culture, antibiotic resistance profiling and antigen agglutination. The local microbiology lab notified the HPT of any positive *Salmonella* results associated with the outbreak by phone in or out of hours.

National reference laboratory processing provided additional serotyping through WGS, with outputs able to be compared with national and international results to determine any linked cases or outbreaks unknown to the local team.

Finally, samples from the facility’s water tank were taken and tested for bacteriological contamination by Scottish Water, the statutory corporation responsible for providing public water supply in Scotland.

## Results

### Outbreak description

Stool samples were tested for 35 symptomatic children and seven adults during the incident; no asymptomatic children were tested. There were a total of 14 confirmed cases of *Salmonella* in children attending the facility, all of the subtype *Salmonella* Saintpaul and with the same genomic sequencing, and one confirmed instance of transmission to an adult family member of a case. Two of the confirmed cases were in siblings who attended different rooms of the facility. The remaining 21 children tested negative for *Salmonella*, as did six staff members who were also tested (the majority of whom were asymptomatic and came forward voluntarily for testing to assist the investigation). Three of the cases were admitted to paediatric assessment units with diarrhoea and dehydration. The remainder of the cases had self-limiting disease which did not require hospitalisation or supportive treatment. None of the cases required or received antibiotic treatment. The facility was attended by a total of 92 children during the outbreak, giving an attack rate of 15.2%.

A significant minority of confirmed child cases (*n* = 5, 35.7%) reported very mild GI symptoms which parents themselves had not felt warranted disclosure to the nursery or HPT, with several describing them as typical of ‘teething nappies’ i.e. the children had stools which were noticeably looser than their normal, but which were not perceived to be severe enough to report even when parents were aware of the ongoing *Salmonella* outbreak. These children were only tested and found to be positive for *Salmonella* when facility staff noted the loose stools and excluded them, with parents asked to submit a stool sample for the child before this exclusion could be lifted. Prior to this enhanced exclusion policy being put in place by the facility, two of the confirmed cases had attended their GP but not had a stool sample requested due to perceived lack of severity by the clinician, and were not tested until a later date when the mild clinical profile associated with the outbreak was highlighted to local GPs by the IMT. Of all confirmed cases, 50% (*n* = 7) had attended the childcare facility either with symptoms or within the 48 h period following recovery of symptoms prior to being formally excluded by the HPT.

**Fig. 1:**
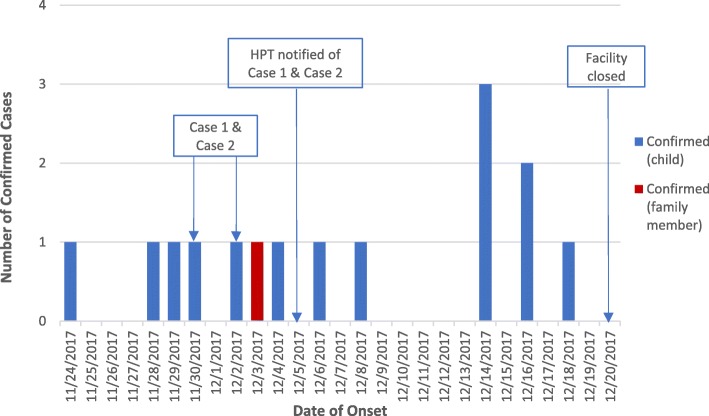
Epidemic curve of confirmed cases by date of onset during the outbreak of *S.* Saintpaul at a childcare facility in Ayrshire, December 2017

The epidemic curve, displaying date of onset for all confirmed cases, did not indicate a single one-off point source of infection (Fig. 1). The epidemiology was instead most in keeping with person-to-person transmission within a shared environment i.e. the childcare facility or, for one case, the home.

**Fig. 2:**
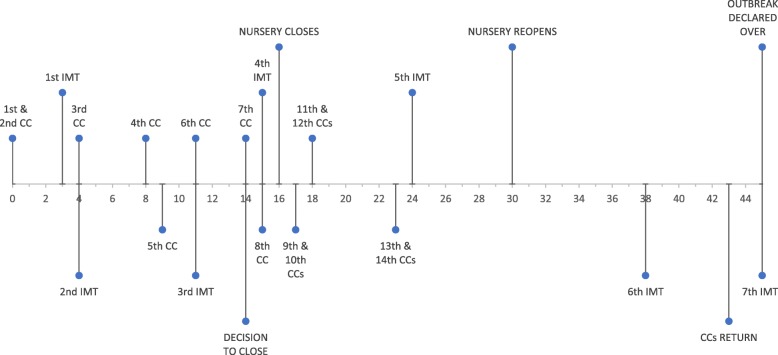
Timeline of case notification and significant events during the outbreak of *S.* Saintpaul at a childcare facility in Ayrshire, December 2017. CC = confirmed case; IMT = Incident Management Team meeting. Day 0 = date first case notified to HPT

The number of confirmed cases in the outbreak increased rapidly within a 4-week period (Fig. 2). Prior to day 14, all cases were confined to one room of the facility. However, the seventh confirmed case and five other possible cases notified on that day were based in different rooms. The remainder of the cases were spread across the three rooms of the facility. The age of confirmed cases ranged from 18 to 58 months, with a mean of 35 months and median of 32 months.

### Laboratory findings

Culture, antibiotic resistance profiling and antigen agglutination were available within 48 h for all samples submitted to the local laboratory. These were identical for each of the 14 confirmed cases, with the organism being fully sensitive to routine antibiotics and agglutinating with the same O4 antigen. Testing of stored water in the facility’s water tank by Scottish Water was performed on day 17 and results were available within 4 days. There was no evidence of microbiological growth in the water sample, indicating that this was unlikely to be the source of infection.

Results from WGS cluster analysis confirmed that each case was of the *S.* Saintpaul serotype with the same Multilocus Sequence Type (MLST50). Including those identified during our outbreak, in total there were 28 isolates of *S.* Saintpaul isolated from patients in Scotland with no history of travel between October 2017 and November 2018. The outbreak strain formed a tight, distinct cluster relative to concurrent non-associated isolates from around Scotland, and no links were found to international isolates when compared with the Enterobase database (containing > 180,000 *Salmonella* genomes).

Of note, WGS results were not available until 2–3 weeks after samples were submitted to the national reference laboratory, meaning in practice the first WGS results did not become available until after key decisions on control measures had been made.

### Public health response

#### Outbreak control measures

Evidence-based control measures were introduced in phases throughout the outbreak according to the most recent risk assessment by the HPT or IMT, and are displayed in Table 2. Risk assessments were updated whenever there was evidence that existing control measures were insufficient to control transmission i.e. when new probable cases were notified with dates of onset after the most recent set of control measures were implemented. Of note, the occurrence of new cases following several satisfactory deep cleans of the facility indicated that a continuous point source from a contaminated surface was unlikely.

**Table 2:** Control measures introduced during the outbreak of *S.* Saintpaul at a childcare facility in Ayrshire, December 2017

Phase of Risk Assessment	Control Measure	Actioned By:
Phase 1 Immediate: Day 0	Exclusion of affected children/staff until 48 h symptom free	Facility
	Removal of sand and water play	Facility
	Deep clean of facility ahead of weekly schedule	Facility
	High temperature washing of fabric toys	Facility
	Disposal of toys unable to be washed	Facility
Phase 2 Initial IMT risk assessment: Day 3	Food safety inspection of facility kitchen	Environmental Health
	Letter to parents and staff re 48-h exclusion for GI symptoms	HPT & Facility
	Site visit to inspect infection control procedures	HPT & HPS
Phase 3 1st reassessment of risk (emergence of mild clinical profile): Day 5	Confirmed cases excluded until negative clearance sample	HPT & Facility
	Interview with external cleaning company to discuss practices	HPS
	Facility social events cancelled	Facility
	Second letter to parents reiterating mildness of symptoms	HPT & Facility
	Text reminder to parents re 48-h exclusion for GI symptoms	Facility
	Parents to sign disclaimer stating children well on attendance	Facility
	Additional deep clean of affected rooms	Facility
Phase 4 2nd reassessment of risk (cases in second room): Day 14	Newly symptomatic children excluded until confirmed negative	HPT & Facility
	Voluntary 2-week closure of facility	Facility
	Further deep clean of whole facility during period of closure	Facility
	Public health exclusion of confirmed cases when facility re-opens	HPT & Facility
	Further testing of negative cases for viral gastroenteritis	Microbiology
	Testing of facility water tank	Scottish Water
	Asymptomatic facility staff to submit stool samples	Facility
	Consider possibility of screening asymptomatic children	IMT
	No mixing of age groups from 7.30 am–8.30 am on re-opening	Facility
	Minimise staff cross-cover between rooms on re-opening	Facility
Phase 5 Final reassessment of risk (facility re-opened): Day 38	Confirmed cases to be excluded until outbreak declared over	HPT & Facility
	Continue without mixing age groups until outbreak declared over	Facility
	Continue increased environmental cleaning until outbreak over	Facility
	On return, confirmed cases to be supervised when handwashing	Facility

There were no new cases following the 2-week voluntary closure of the facility. Confirmed cases continued to be supervised when handwashing until 2 weeks after the outbreak was declared over, at which point it was felt this final control measure was no longer indicated.

#### Environmental investigations

The facility’s routine infection control protocols were scrutinized by the IMT during their first meeting and found to be satisfactory, including those protocols relating to food preparation, toileting and environmental cleaning. Both site visits including the food safety inspection and review of menus were satisfactory, with only very minor recommendations made, and no obvious continuous point source of infection found. During a telephone interview, the infection control specialist from HPS was satisfied with the cleaning practices of the external cleaners, with the only recommendation made being a switch from reusable to disposable cloths during the outbreak.

Overall, there was no evidence of any failure on the part of the facility in maintaining adequate infection control procedures either before or during the outbreak.

#### Epidemiological investigations

Interviews with parents or guardians of confirmed cases and completed gastrointestinal surveillance forms indicated no epidemiological link other than the childcare facility, with the exceptions of the one sibling pair and one secondary case in an adult family member. There were several reports of children being sent to the facility with mild GI symptoms which parents did not feel merited disclosure, despite knowledge of the ongoing outbreak. Difficulty finding alternative childcare provision was frequently discussed as a motivating factor in resisting the exclusion of symptomatic children who parents did not perceive to be particularly unwell.

## Discussion

We report a relatively small *Salmonella* Saintpaul outbreak linked to a childcare facility, with 15 confirmed cases in total who all made a full recovery. The outbreak proved unusually challenging to manage due to its particularly mild and almost subclinical presentation in many confirmed cases, with over a third having only slightly loose stools compared with their usual and being otherwise well, despite several children becoming unwell enough to require hospital attendance for dehydration. The standard evidence-based control measures for *Salmonella* were insufficient to control transmission and a pragmatic and iterative approach to incident management was required, with gradual scaling up of control measures to the eventual voluntary closure of the facility (Table 2). This was relatively straightforward to arrange due to the co-operation of the facility and the fact the closure spanned an existing holiday period, but would have been more problematic in other circumstances.

From epidemiological investigations, the most likely mode of transmission was person-to-person spread between children within the childcare facility. There was no evidence of a single or continuous point source of *S.* Saintpaul within the facility despite consideration and investigation of potential food, water or environmental contamination. This is the first *S.* Saintpaul outbreak reported with this as the most likely mode of transmission, as all previous reports in the literature involved contamination of food or environment, [15, 16] most often raw fruit or vegetables [17–20] or water [21, 22].

Salmonellosis in general is most often conceptualized as a foodborne illness when investigating school or pre-school childcare facility outbreaks, [6, 23] or less commonly linked to specific events such as animal handling [24] or sand play [12]. In previous pre-school outbreaks of *Salmonella* where person-to-person spread was felt to be a contributing factor, this was found to be secondary to poor hygiene practices among staff, [25, 26] though direct spread from one infected child to another (particularly among those able to crawl) was postulated as a potential secondary mechanism [27].

Despite significant interrogation of the infection control protocols and procedures at our facility, we could find no major faults which would account for the ongoing transmission. Therefore, we judge that transmission in this case was most likely due to confirmed and unknown cases attending the facility while symptomatic, indicating the importance of exclusion of symptomatic children until 48 h symptom free in the current guidance [13]. We based this conclusion on the detailed attendance history we gathered for all confirmed cases, which indicated that half of them had attended the facility in conditions which would be against standard public health advice.

One previous report of a *Salmonella* Paratyphi outbreak in a boarding school where cases were surveyed found individual poor handwashing by children to be independently associated with infection, second only to drinking unboiled water from a potentially contaminated source [11]. It may be that the role of individual hygiene practices among children has been underestimated during previous childcare-related outbreaks, particularly those involving very young children. We considered also the possibility that thumb-sucking among affected children may have contributed to the unexpectedly high levels of child-to-child transmission in our outbreak. Though we were unable to gather sufficient data to formally test this hypothesis in our sample, we note it has been suggested as a possible mediator during similar childcare facility outbreaks [27].

There is evidence that the bacterial dosage received by individuals infected with *Salmonella* may influence both incubation period and symptom severity [4, 28]. Given that person-to-person spread of *Salmonella* would result in a particularly small infective dose [27] it is possible that this mode of transmission is responsible for the unusually mild symptoms described by parents of many confirmed cases in our outbreak. In future outbreaks where person-to-person transmission is considered a possibility due to poor hygiene among either young children or staff, active case finding using communications which highlight the potential for milder symptoms may be useful.

Early communication between the childcare facility and HPT uncovered a marked disparity between the symptoms being disclosed to facility staff and public health professionals, with parents and guardians more likely to disclose symptoms to the HPT, often only once a case was confirmed. On questioning, this lack of disclosure was often reported to be due to concerns about the availability of alternative childcare arrangements and/or difficulties in taking leave from employment.

This is in keeping with recent qualitative work exploring parental decision-making when choosing whether to send children with respiratory infections to childcare facilities, which found that this involved a complex interaction between the perceived severity of the child’s illness, the policies of the childcare facility, and personal circumstances including possible work absences and financial penalties [29]. In our case, once this issue became known to the IMT both facility staff and HPT members were able to pre-empt conversations about these emotive issues with parents, taking time to have detailed conversations about the concerns and expectations of the parents around disclosure, following which there was some improvement in co-operation.

Finally, in our outbreak WGS results were informative in linking the cases to each other and excluding any link to international outbreaks post-event, as has been reported in many similar studies [30–32]. However, they were not able to inform the active epidemiological investigation or influence decisions around necessary control measures due to unavoidable technical delays in receiving the results. While these techniques continue to hold promise in confirming epidemiological links during ‘live’ outbreaks in future, [33] in our setting until typing is available with the same speed as conventional results they are unlikely to influence public health practice during short localised outbreaks.

## Conclusions

Child-to-child spread should be considered a potential route of transmission during *Salmonella* outbreaks in childcare facilities, and may result in unusual presentations or epidemiological patterns. Standard infection control measures may not be sufficient to control outbreaks of *Salmonella* with mild clinical features in childcare settings, particularly where there are concerns about the degree of parental disclosure. Proactive communication with parents exploring barriers and concerns may increase disclosure of symptoms. In Scotland and other countries with similar systems, the use of whole genome sequencing for typing is only likely to be useful in confirming epidemiological links during an ongoing outbreak if results can become available in a timely manner, or in more dispersed outbreaks to identify cases who are not linked by a known common location.

## Data Availability

The datasets generated and/or analysed during the current study are not publicly available due to their potentially disclosive nature, but de-identified data may be made available by the corresponding author on reasonable request.

## Abbreviations

GI: Gastrointestinal
GP: General Practitioner
HPS: Health Protection Scotland
HPT: Health Protection Team
IMT: Incident Management Team
MLST: Multilocus Sequence Type
MST: Minimum Spanning Tree
WGS: Whole Genome Sequencing
